# General and Postbariatric Nutritional Knowledge among Patients Undergoing Bariatric Surgery

**DOI:** 10.1155/2019/6549476

**Published:** 2019-12-13

**Authors:** Areej Alkhaldy, Banan Alshehri, Nouf Albalawi, Farah Alsaady, Renad Alfarshooti, Wisam Jamal, Abdulmalik Altaf, Ashraf A. Maghrabi

**Affiliations:** ^1^Clinical Nutrition Department, Faculty of Applied Medical Sciences, King Abdulaziz University, Jeddah, Saudi Arabia; ^2^Department of Surgery, Faculty of Medicine, University of Jeddah, Jeddah, Saudi Arabia; ^3^Department of Surgery, Faculty of Medicine, King Abdulaziz University, Jeddah, Saudi Arabia

## Abstract

**Purpose:**

The prevalence of obesity and the number of bariatric surgeries are increasing in Saudi Arabia. Studies evaluating nutritional knowledge, especially in Middle Eastern countries, are limited. Therefore, this study was conducted to examine the general and postbariatric nutritional knowledge related to dietary recommendations among patients undergoing bariatric surgery.

**Patients and Methods:**

In a cross-sectional study, 112 patients aged 18–65 years, of both genders, were recruited from the Surgical Clinics at King Abdul-Aziz University Hospital in Jeddah, Saudi Arabia. Patients' knowledge pertaining to general nutrition and consumption after bariatric surgery was assessed in relation to dietary recommendations, using a preoperative questionnaire.

**Results:**

The mean general nutrition knowledge score was 42 of a maximum of 85 points (50%). Approximately 40% and 60% of patients were classified with a low and medium level of nutritional knowledge, respectively. Postbariatric nutritional knowledge among patients was very low (mean: 16/81 points). The level of education was correlated with patients' body mass index (*p*=0.045) and the general nutritional knowledge total score (*p*=0.05).

**Conclusion:**

General and postbariatric nutritional knowledge among Saudi bariatric patients is currently insufficient. A multicenter study involving a larger sample size with different sociodemographic characteristics is warranted to confirm these findings. The purpose of such a study would be to determine the nutritional knowledge of patients undergoing bariatric surgery and inform the implementation of educational strategies.

## 1. Introduction

Obesity is a major public health concern in the Kingdom of Saudi Arabia, where one in three adults are obese and at least one in 10 adults have morbid obesity [1, 2]. This condition is a risk factor for a range of diseases (e.g., diabetes, cardiovascular diseases, and cancer-causing morbidity and mortality) that adversely impact the health of the Saudi population [3].

Bariatric surgery has become increasingly popular as the most effective treatment for morbidly obese individuals compared with other weight loss strategies, such as dietary restriction, physical activity, and behavioral therapy [4]. This is attributed to the significant weight loss experienced and overall improvement in obesity-related comorbidities [5, 6]. However, it is typically considered as the last option for the treatment of patients with morbid obesity, owing to the complications that may arise postoperatively [7].

According to the guidelines established by the American Society for Metabolic & Bariatric Surgery, patients should undergo appropriate nutritional evaluation, including measurement of micronutrients, prior to any bariatric surgical procedure [8]. Moreover, these guidelines state that patients should undergo an evaluation of their ability to incorporate nutritional and behavioral changes prior to and after bariatric surgery [8]. Noncompliance of bariatric patients to the dietary recommendations has been associated with the risk of developing postoperative complications [9]. In particular, nutritional deficiencies in protein, iron, vitamin B12, folate, calcium, fat-soluble vitamins, and other micronutrients are a concern following bariatric surgery [10, 11]. Furthermore, noncompliance to dietary recommendations leads to rapid weight regain or failure to reach the appropriate weight after bariatric surgery [12]. Therefore, it is essential to evaluate the general nutritional knowledge of bariatric patients regarding appropriate sources of nutrients, selection of daily food, and the diet-disease relationship to identify knowledge gaps related to dietary intake and habits that may have contributed to their obesity. This information will assist in planning appropriate educational intervention strategies that may improve knowledge and aid adherence to the dietary and lifestyle changes necessary for the successful long-term outcome of bariatric surgery [13, 14].

The short-term outcome of surgery is also dependent on patient adherence to the dietary recommendations after surgery, which includes advice on diet progression, dietary-related behaviors, use of protein and other micronutrient supplements, and nutritional therapy for common gastrointestinal symptoms [14]. Therefore, the evaluation of postbariatric nutritional knowledge is crucial to determine patients' understanding of the specific dietary changes required following bariatric surgery to minimize the risk of postoperative complications and assist bariatric surgical intervention achieve its goals.

In Saudi Arabia, bariatric surgery is the preferred option with 15,000 operations performed annually [1]. However, current research regarding bariatric surgery is dominated by studies involving Western populations, with a paucity of research evaluating the nutritional knowledge of bariatric patients. Therefore, the aim of the present study was to examine the general and postbariatric nutritional knowledge related to dietary recommendations among patients undergoing bariatric surgery. We hypothesized that postbariatric nutritional knowledge related to dietary recommendations is not sufficient among such patients.

## 2. Material and Methods

### 2.1. Study Design

A cross-sectional study was performed at King Abdul-Aziz University Hospital (KAUH) in Jeddah, Saudi Arabia. Ethical approval was obtained from the Faculty of Medicine Research Committee at King Abdul-Aziz University (reference no. 74-17). All patients provided written informed consent.

### 2.2. Participants and Recruitment

The study included bariatric patients randomly recruited from the outpatient surgical clinics at KAUH. The inclusion criteria for participants were as follows: age between 18–65 years, male or female patients, a body mass index (BMI) of ≥35 kg/m^2^, and scheduled to undergo bariatric surgery.

### 2.3. Sample Size Calculations

Currently, there are no previous studies conducted in Saudi Arabia investigating the nutritional knowledge of bariatric patients or that are similar in nature to be used as a reference. Therefore, we performed the sample size calculation based on the KAUH statistics regarding bariatric surgeries from 2018 using the online Epi Info sample size calculator (Division of Health Informatics & Surveillance, and Centre for Surveillance, Epidemiology & Laboratory Services) [15]. The KAUH statistics showed that approximately 190 patients underwent bariatric surgeries. Therefore, the sample size was calculated by considering 50% of patient knowledge regarding the general and postbariatric surgery diet phases, a confidence level of 90%, a margin error of 5%, and a design effect of 1. Hence, the total calculated sample size was 112 participants.

### 2.4. Study Instrument

The study instrument consisted of three sections. Section one (comprising six questions) was used to collect background and sociodemographic information, including age, gender, educational level, household income, weight, and height (used to calculate the BMI). Section two assessed the general nutritional knowledge of the bariatric patients using a validated General Nutrition Knowledge Questionnaire (GNKQ) adapted from Kliemann et al. [16], modified, and translated from English to Arabic using the Brislin back-translation method [17, 18]. For pilot testing, the questionnaire was shared with three Ph.D. holders in the Clinical Nutrition Department and Surgery Department. The questionnaire was subsequently revised based on their responses. Appropriate modifications were made for the Saudi participants of this study, to remove references to food and drink items not culturally relevant to Saudi Arabia (i.e., canned soup, plantains, and alcohol). As a result of these modifications, the final questionnaire was composed of a total of 47 questions (85 points).

The GNKQ consisted of four parts. Part one (8 questions, 17 points in total) was used to assess participants' awareness regarding dietary recommendations; part two (10 questions, 34 points in total) was used to measure participants' awareness of food groups and the nutrients they contain; part three (13 questions, 13 points in total) was used to assess participants' knowledge regarding healthy food choices; and part four (16 questions, 21 points in total) was used to assess participants' awareness of associations between diet, disease, and weight. The GNKQ was scored according to Kliemann et al. [16] For this study, the level of a patient's nutritional knowledge was classified as follows: participants with a total point score <50%, 50–75%, and >75% of the possible point total of the assessed area were classified as “low nutritional knowledge,” “medium nutritional knowledge,” and “high nutritional knowledge,” respectively. Section three assessed knowledge regarding diet phases and requirements after surgery, as well as surgery-related complications, using a validated nutrition knowledge questionnaire.

The Eating After Bariatric Surgery (EABS) questionnaire was adapted from Taube-Schiff et al. [19], modified, and translated from English to Arabic using the Brislin back-translation method [17, 18]. The questionnaire contained 12 questions (seven questions are multiple-choice, three questions fill-in-the-blank questions, and two short answer questions). The scoring of the EABS questionnaire was according to Taube-Schiff et al. [19]. The permission to use the EABS was obtained from Springer Nature via Copyright Clearance Centre (license no. 4607611459606).

### 2.5. Statistical Analysis

Data were analyzed using Microsoft Office 365 Pro Plus and SPSS version 23.0 (IBM Corp., Armonk, NY, USA). Data from the GNKQ and EABS were presented using descriptive statistics. The Pearson correlation was used to identify relevant associations among the study variables of interest. Statistical significance was determined by a *p* value <0.05.

## 3. Results

### 3.1. Subjects Characteristics

Table 1 summarizes the demographics of the study sample. A total of 112 patients were recruited in this study. The sample predominantly included female patients (59%, *n* = 66), with a mean age of 37 years. Nearly half of the patients were classified in the lowest category of income (44%), and 57% were educated to at least Bachelor's degree level. The correlation among the main study variables was analyzed by the Pearson correlation, indicating that the educational level of patients was negatively correlated with their BMI (*R*^*2*^ = −0.190; *p*=0.045).

### 3.2. GNKQ

Table 2 shows the descriptive statistics and nutritional knowledge level for the total score and each part of the GNKQ in section two of the questionnaire. The mean total score of the patients was 42/85 points (50%). Using the percentile thresholds defined in the methods, the number of patients classified with lower, medium, and high nutritional knowledge was 45 (40%), 66 (59%), and one (female, 21 years old, with a Bachelor's degree), respectively. There were no significant differences found between male and female patients. The score of part three (healthy food choices) yielded the lowest values.

### 3.3. Correlation between the Total Nutritional Knowledge Score and Main Study Variables

The relationship between the GNKQ total score and main study variables (i.e., age, gender, education, income, and BMI) was analyzed using the Pearson correlation. A relationship was found only between the educational level of patients and nutritional knowledge (*R*^*2*^ = 0.186; *p*=0.05).

### 3.4. EABS Questionnaire

Table 3 shows the descriptive statistics of the 12 questions and their total score. All patients were rated to have very low knowledge (mean: 16/81 points). There were no significant differences found between male and female patients. Overall, the level of knowledge among patients regarding the recommendations for EABS was low (mean: 16/81 points, 20%), and a mean of zero points scored for questions regarding signs of dumping syndrome, maximum amount of food that should be eaten at one meal after healing, and protein that should be eaten after weight loss surgery.

## 4. Discussion

This study was performed to examine the general and postbariatric nutritional knowledge related to dietary recommendations among bariatric surgery patients attending the outpatient surgical clinic in KAUH. Despite the high prevalence rate of obesity and the number of bariatric surgeries in Saudi Arabia [1, 2], no studies have previously examined the nutritional knowledge among bariatric patients. Therefore, the data acquisition from this study is an important first step in understanding the current status of Saudi bariatric patients' nutritional knowledge and literacy. The results provide a baseline for estimating the status of nutrition knowledge among presurgery bariatric patients and will inform areas of particular knowledge deficit that can feed into the planning of targeted educational sessions to improve adherence to postoperative dietary recommendations and lifetime dietary changes.

In the present study, we found that approximately 40% of study participants were assessed as having a low level of general nutrition knowledge, while 59% had a medium level of knowledge. Only one individual (female, 21 years old, with a bachelor's degree) was assessed as having a high level of nutritional knowledge. The area of knowledge with the greatest deficit was in healthy food choices (section 2, part 3). The questions in this section assess the patient's knowledge about ‘traffic lights' food labelling system, nutrition claims, ingredient lists and calorie content, preparation methods, and healthy meal choices. This nutritional knowledge could be a contribution factor for obesity in this studied group, as food choices determine energy and nutrient intake [20, 21]. Although food choices are influenced by many components, including education, cultural, and economic factors, in this study, we only detected a correlation between patients' BMI and general nutritional knowledge. The greater prevalence of obesity among the population with a lower education has been reported previously [22]. However, Al-Ghamdi et al. did not report education as a significant predictor of obesity among Saudi obese individuals [23]. There is a need for more studies in Saudi Arabia to explore the association between obesity and the level of education among the Saudi population. In the biethnic study and Centers for Disease Control and Prevention report, the level of education was found to be dependent on the ethnicity and differed between high-income and low-income countries [24, 25]. For example, the level of education was a significant predictor of high weight for Americans but not for the Japanese [25]. Moreover, there were no differences found in the prevalence of obesity according to the level of education among non-Hispanic Asian women and men [24]. There is limited evidence in Saudi Arabia regarding the relationship between obesity and the level of education. Therefore, it is important to consider this relationship in future studies, especially with the globalization of the food industry and high physical inactivity (67%), with 88% of leisure time spent in inactivity among Saudis [26]. In addition, the low-nutritional literacy could be due to the absence of nutritional education and counseling [27]. Nutritional education is commonly used to help change human behaviors and is provided with the expectation that greater knowledge will improve dietary practices and healthier food choices [27]. Research has revealed an increase in the consumption of the fruit and vegetable in adults with improved knowledge [28], and that equipping university students with greater awareness of healthy eating guidelines make better food choices [29]. In the current study, 32% of the participants had diabetes, and 28% had hypertension. Therefore, education is very important for obese individuals to obtain successful outcomes of surgery and control the chronic diseases that accompany obesity.

Similarly, all patients scored poorly on the postbariatric nutritional knowledge questionnaire, even though they had already been assigned to surgery and more than half were educated to at least Bachelor's degree level. One potential reason for this knowledge deficiency could be related to the current Saudi hospital system, which only arranges for the patient consultation with a dietitian after the bariatric surgery had been scheduled which could be due to lack of educational protocol, long waiting list, or patient's compliance issues with clinic appointments. This is supported by Alia et al. who reported that the majority of their participants correctly responded to the knowledge question related to the clear liquid diet stage, as the patients are usually given this information during their hospital stay [30]. Our patients were recruited from the outpatients' clinic before the admission process, which usually provided the patients with the surgery information sheet, containing nutritional advice. Further study is needed to evaluate whether this information sheet provides sufficient nutritional education for the patients, as well as to determine the best strategies with which to deliver the information, in order to maximize the patient's understanding and adherence.

The findings of this study indicate the importance of including a registered dietitian in the multidisciplinary team to provide the patients with the necessary nutritional information before the surgery, and in the short and long-term postbariatric surgery follow-up [31]. The positive role of the registered dietitian has been reported to bring value to the healthcare team and delivery models by improving the outcomes for patients with a wide variety of medical conditions, including bariatric patients [8, 32]. Many studies have indicated the importance of nutritional management, education, and counseling for bariatric patients by registered dietitians (RD) [33]. For example, presurgery nutrition education classes have been shown to significantly improve patients' postbariatric nutritional knowledge, particularly when conducted close to the date of surgery [19]. Recently, a study in the United Arab Emirates reported the need to develop and implement nutritional counseling strategies to improve the adherence of bariatric patients to the postoperative dietary recommendations [30]. Therefore, it is important to include RD in the multidisciplinary team to communicate this information to patients both before and after surgery to improve surgical outcomes.

Similar to most cross-sectional studies, our study was characterized by limitations. Firstly, the mode of questionnaire administration (interviewer-administered questionnaires) may have had an impact on data quality and the answers provided by patients. The interviewer-administered questionnaires could create the potential for the interviewer to intentionally or unintentionally influence the answers as it may yield more motivation, as well as better and more accurate responses from respondents [34]. In addition, it may increase the social desirability bias [34]. However, this limitation is common in most studies because there are advantages and disadvantages to each mode of questionnaire administration. Different modes of questionnaire administration have different inherent biases and currently, there is no clearly superior method. Future studies need to consider the training, guidelines, and monitoring for administration of questionnaires to ensure a standard approach to data collection by the investigators and minimize the disadvantages [34]. Also, the analysis of responses by multiple interviewers can check the data for interviewer bias [34]. Secondly, the data from this study cannot yet be widely generalized as the recruitment was from a single site (university hospital), which is also considered a busy site with a large proportion of low-income patients. A multicenter study is now needed, using a larger sample size with different sociodemographic characteristics, in order to fully evaluate the current nutritional literacy of Saudi bariatric surgery patients. In addition, there is a need to explore the association between the preoperative nutritional knowledge scores with postoperative general and nutritional-related complications, the efficacy of a structured nutritional education on postoperative nutritional deficiency, and the weight-related outcomes among Saudi bariatric patients.

## 5. Conclusion

This study reveals the fundamental need to improve nutritional literacy among bariatric patients undergoing surgery. The aim of such an intervention is to increase adherence to the dietary recommendations postbariatric surgery and empower patients with sufficient knowledge to make the necessary lifelong dietary changes to avoid nutritional deficiency. The data of this study may be used as a baseline for future studies to optimize educational strategies by investigating the impact of time on nutritional knowledge retention among bariatric patients.

## Figures and Tables

**Table 1:** Demographic and anthropometric data (*n* = 112; *F* = 66; *M* = 46)^*∗*^.

	Mean	SD
Age (years)	37.4	11.1
Height (m)	1.6	0.1
Weight (kg)	126.6	28.0
BMI (kg/m^2^)	46.8	8.3

BMI categories	*n*	%

Obese class 1	5	4
Obese class 2	15	13
Obese class 3	92	82

Income	*n*	%

<5,000	49	44
5,000 to 10,000	33	29
10,000 to 15,000	19	17
>15,000	11	10

Education	*n*	%

Primary	6	5
Secondary	27	24
High school	15	13
Bachelor	55	49
Higher education	9	8

Medical history	*n*	%

Gastroenterology diseases	9.0	8.0
Cardiovascular diseases	5.0	4.5
Diabetes	36.0	32.1
Hypertension	31.0	27.7

^*∗*^ *M* = male, *F* = female, and *n* = number. The BMI categories are defined as follows: obese class 1 (BMI, 30.0–34.9 kg/m^2^), obese class 2 (BMI, 35.0–39.9 kg/m^2^), and obese class 3 (BMI, ≥40 kg/m^2^).

**Table 2:** Bariatric patients' GNKQ scores and general nutritional knowledge level (*n* = 112).

Knowledge domain (max possible score)	Mean	SD	Min	Max	Lower knowledge (<50% percentile)	Medium knowledge (50–75% percentile)	Higher knowledge (>75% percentile)
					*n* (%)	*n* (%)	*n* (%)
Part 1: dietary recommendations (17)	9	3	1	14	38 (34)	63 (56)	11 (10)
Part 2: food groups (34)	16	6	0	28	52 (46)	55 (49)	5 (4)
Part 3: healthy food choices (13)	6	2	0	11	57 (51)	46 (41)	9 (8)
Part 4: diet, disease, and weight associations (21)	10	4	0	18	50 (45)	54 (48)	8 (7)
**Total: nutrition knowledge score (85)**	**42**	**13**	**1**	**67**	**45 (40)**	**66 (59)**	**1 (1)**

**Table 3:** Bariatric patients' EABS score for postbariatric nutritional knowledge (*n* = 112).

No	Questions (max possible score)	Mean	SD	Min	Max
1	Knowledge about the liquid diet (6)	1	1	0	5
2	Knowledge about the pureed diet phase (4)	2	1	0	5
3	Knowledge about the soft diet phase (5)	2	1	0	4
4	Knowledge about the high protein diet (9)	3	2	0	8
5	Knowledge about vitamins and minerals (3)	1	1	0	5
6	Knowledge about the signs of dumping syndrome (1)	0	1	0	6
7	Knowledge about the maximum amount of food that should be eaten at one meal after healing (1)	0	0	0	1
8	Knowledge about maximum amount of time that one should go without eating after weight loss surgery (5)	1	2	0	8
9	Knowledge about the recommended amount of fluid that should be drunk after healing (5)	1	2	0	9
10	Knowledge about the amount of protein that should be eaten after weight loss surgery (5)	0	1	0	5
11	Knowledge about preventing nausea and vomiting (20)	2	4	0	15
12	Knowledge about preventing dumping syndrome (15)	1	3	0	15
	**Total questions (81)**	**16**	**8**	**3**	**39**

## Data Availability

The data used to support the findings of this study are available from the corresponding author upon request.
